# Vaccine Hesitancy towards COVID-19 Vaccination: Investigating the Role of Information Sources through a Mediation Analysis

**DOI:** 10.3390/idr13030066

**Published:** 2021-08-12

**Authors:** Chiara Reno, Elisa Maietti, Zeno Di Valerio, Marco Montalti, Maria Pia Fantini, Davide Gori

**Affiliations:** Department of Biomedical and Neuromotor Sciences, Alma Mater Studiorum—University of Bologna, 40126 Bologna, Italy; chiara.reno@studio.unibo.it (C.R.); zeno.divalerio@studio.unibo.it (Z.D.V.); marco.montalti7@studio.unibo.it (M.M.); mariapia.fantini@unibo.it (M.P.F.); davde.gori4@unibo.it (D.G.)

**Keywords:** SARS-CoV-2, COVID-19, pandemic, social media, mediation analysis, structural equation modelling, survey, vaccine hesitancy, vaccine acceptance, information sources

## Abstract

Mass vaccination campaigns have been implemented worldwide to counteract the SARS-CoV-2/COVID-19 pandemic, however their effectiveness could be challenged by vaccine hesitancy. The tremendous rise in the use of social media have made them acquire a leading role as an information source, thus representing a crucial factor at play that could contribute to increase or mitigate vaccine hesitancy, as information sources play a pivotal role in shaping public opinion and perceptions. The aims of the study were to investigate if information sources could affect the attitude towards COVID-19 vaccination and if they could act as a mediator in the relationship between individual characteristics and vaccine hesitancy. A cross-sectional online survey was conducted by a professional panellist on a representative sample of 1011 citizens from the Emilia-Romagna region in Italy in January 2021. A mediation analysis using structural equation modelling was performed. Our results show how social media directly or indirectly increases vaccine hesitancy towards COVID-19 vaccination, while the opposite effect was observed for institutional websites. Given the global widespread use of social media, their use should be enhanced to disseminate scientifically sound information to a greater audience to counteract vaccine hesitancy, while at the same time continuing to promote and update institutional websites that have proven to be effective in reducing vaccine hesitancy.

## 1. Introduction

The coronavirus disease 2019 (COVID-19) pandemic caused profound changes in daily life and shifted collective priorities, with incalculable and in some cases unforeseeable damage that has yet to fully unravel, and in mid-2021, after about a year and a half since its onset, is still a threat to individual health and society. As the efforts to devise course-changing therapies have not yet come as far as to represent a viable strategy in containing the pandemic, most countries have mainly relied on mass vaccination campaigns to curb its harmful consequences. This strategy has been met with some success, as the effect of growing rates of immunized people have appeared to have had an impact on epidemiological curves in Israel and the United Kingdom (UK) and on disease severity in the United States (US) [1,2,3]. As the purpose of COVID-19 vaccination campaigns turned from containing an emergency to providing a reliable and constant defence against further menace to people and health systems, reaching and maintaining high rates of immunization became of the utmost importance. In this light, vaccine hesitancy has the potential to greatly complicate the attempts to reach the estimated threshold for population level immunity [4].

Although particularly relevant in this historical moment during which new vaccines have been developed and licensed at an unprecedented speed, vaccine hesitancy has been a long-standing major public health concern as old as vaccination itself [5]. In recent years, vaccine hesitancy has reached worrying proportions, slowing down attempts to reach high rates of immunization [6] and reducing vaccination coverage to the extent of causing outbreaks of infectious diseases which had been under controls for years, as is in the case of several measles outbreaks in the UK [7] and the US [8], inducing the WHO to define it as one of the major global health concerns in 2019 [9].

In 2015, the World Health Organization defined vaccine hesitancy as a “delay in acceptance or refusal of safe vaccines despite availability of vaccine services” and the wide variety of reasons behind vaccine hesitancy have been summarized by the Scientific Advisory Groups of Experts (SAGE) Working Group on Vaccine Hesitancy in the three Cs framework (“Confidence”, “Complacency”, and “Convenience”) [10], which effectively encapsulates different determinants that could foster vaccine hesitancy, and was subsequently expanded to the five Cs by adding “Context” and “Communication” [11].

Among other factors, a lack of trust in political and scientific institutions has been shown to lower vaccine acceptance [12,13,14]; similarly, the disappearance of the most harrowing consequences of many infectious diseases, especially from Western societies, has lowered risk perception and has thereby lowered vaccine uptake [15]. Income level and socioeconomic status were identified as factors affecting vaccine acceptance [16]; a low educational level and health illiteracy have been linked to a reduced vaccine confidence level [16,17,18], and they may hinder access to information on the benefits of immunization and on the detrimental consequences of infectious diseases. Moreover, structural factors such as health inequalities, systemic racism, and barriers to access could contribute to vaccine hesitancy and lead to decreased uptake [19].

The contribution of information sources to vaccine hesitancy has been known for a while, as their role is not only to passively transfer information to the public, but to select what is relevant for discussion by reporting facts with specific timing and emotional framing. In particular, the emotional context within facts has been shown to affect opinions considerably, with emotionally charged facts and personal narratives having a potential advantage over objectively and scientifically presented information [20,21]. Such dynamics have been shown to considerably affect vaccine hesitancy, as has been exemplified by the measles, mumps, and rubella (MMR) vaccine controversy in the UK in 1999, which caused a sharp drop in immunization rates (from a national level of 80% to a level lower than 60% in some areas) and consequent measles outbreaks [22]. The role of traditional media in distorting the public perception of scientific debate has since been confirmed [23,24]. A recent literature analysis determined the important role of information in determining vaccine propensity. In particular, trust in the information source, the understandability of information, and how it is received by individuals are factors linked to the effect of information on vaccine hesitancy [25].

Among the information sources, social media became central to the scientific and political debate, revolutionizing the ways of communicating, being easily accessible, and rapidly connecting millions of individuals all around the world, accelerating the exchange of information, thus determining a shift from traditional media and becoming a primary and predominant informal information source [26] that millions of people rely on.

Social media have been recognized as a potentially useful tool for health education and promotion, and many public health organizations use social media to reach the public [27]. Moreover, they could be useful for the dissemination of innovations and research findings, fostering collaboration throughout digital engagement [28]. It is widely recognized that social media can play a role during infectious diseases outbreaks in increasing awareness of the disease and its prevention by providing updates and in shaping people’s risk perception, thus potentially impacting decision making and behaviours. In fact, social media played a central role in informing the public during outbreaks such as the H1N1 outbreak in 2009 and the Ebola outbreak in 2014 [27,29]. Furthermore, they can play a helpful role in enhancing public health surveillance through real-time reporting to counteract the spread of infectious diseases, and researchers are increasingly incorporating social media data into mathematical models to describe infectious diseases dynamics [28,30].

A further increase in their use has been observed during the COVID-19 pandemic, with unprecedented peaks linked on one side to lockdown and restrictive measures, and on the other side to the urgent need of rapidly communicating the enormous amount of research generated in a short time to quickly react to the emergency, giving rise to real-time global communication among scientists on a scale never seen before [31,32]. Emblematic in this sense is the dissemination of the genomic sequence of SARS-CoV-2 through Twitter [32].

However, considering that information is based on user-generated content, there are many inherent risks in these sources of information, from information overload, inaccuracy, and the dissemination of information that is not carefully reviewed or of low-quality, to misinformation and the spread of conspiracy theories [27,28].

Such issues are likely to have played a role in shaping the current state of the discussion on vaccines on social media. Social media can increase the magnitude of influence of antivaccination groups and can contribute to new methods of self-organisation and empowerment for online communities that debate against or in favour of vaccines [33]. Content regarding vaccines published on social media expressing hesitant views have an apparent advantage in terms of shares if compared to neutral or positive stances [34,35]. Moreover, the exposure to such content and the use of social media as a primary source of information have shown to be able to effectively shift views of readers towards those of hesitancy [36,37,38].

The combination of this skewed environment and the speed at which information is conveyed and spread across social media has produced tangible effects of vaccine uptake, which has proven to be extremely volatile in response to events regarding vaccines [20]. These effects are sometimes actively and consciously engendered with a malicious intent [39]. There is also some evidence showing how the wide reach and low access cost of social media have made them an instrument in influencing debate with the conscious aim of destabilizing a country, as “bots” and “trolls” have become commonplace across all platforms [39].

Our study aims to provide some clarity and insight in this ever-changing environment by investigating the relationship between sources of information and vaccine hesitancy towards COVID-19 vaccination. Our hypothesis is that the type of information source used could affect attitudes towards COVID-19 vaccination and could mediate the relationship between individual characteristics and vaccine hesitancy. Specifically, considering the abovementioned inherent risks of information disseminated through social media, such as inaccuracy or low-quality, and that the content spread through social media has been shown to be predominantly against vaccination, we hypothesize that the use of social media could increase vaccine hesitancy. Assessing the role of information sources could provide stakeholders and institutions concrete elements for a more effective use of media and chiefly of the yet untapped potential of social media.

## 2. Materials and Methods

### 2.1. Study Design and Data Collection

Our study was a cross-sectional online survey. A professional panel provider (Doxa S.p.A.) recruited a representative random sample of citizens of the Emilia-Romagna region using a quota-based sampling strategy to ensure representativeness by sociodemographics and geographical distribution. Participant age ranged from 18 to 70 years. The survey was implemented from 19 January to 26 January 2021 after the start of the SARS-CoV-2 vaccination campaign by healthcare professionals. The Doxa data management was performed in accordance with the General Data Protection Regulation of the EU.

### 2.2. Questionnaire

The questionnaire was adapted by a survey tool used in the US as previously described [17,40]; the questions focused on socio-demographic characteristics, comorbidities, history of vaccination refusal, perceived risk of infection, likelihood of accepting the COVID-19 vaccination, and the sources used to obtain information on COVID-19 vaccines. An English version of the survey instrument can be found in the Supplementary Materials (see Supplementary Materials, Questionnaire instrument).

Cognitive testing was conducted prior to full implementation. Feedback was used to revise the questionnaire. This survey was designed to be completed in approximately ten minutes.

Respondents were asked about their willingness to get vaccinated if a COVID-19 vaccine had been offered to them free of charge in the months ahead. The answer options were: “very likely”, “somewhat likely”, “not sure”, “not in the next two months but would consider it in the future”, “somewhat unlikely”, “very unlikely”. For the purpose of this analysis, the responses were dichotomized into two categories (1 = “Hesitant” including “very unlikely/somewhat unlikely/not sure/not in the next months but I would consider it in the future” and 0 = “Confident”, including “very likely/somewhat likely”).

Independent variables included socio-demographics such as age, gender, educational level, and family income. Other independent variables were past vaccination refusal, risk perception of contracting the disease or infecting others, and the presence of comorbidities associated with a clinical risk of severe consequences from COVID-19. In particular, risk perception was measured by the level of concern of contracting COVID-19 at work or outside of the work environment and of infecting family members or friends (options: “not at all”, “a little”, “somewhat”, “much concerned”). Comorbidities included the health conditions most frequently associated with severe COVID-19 disease or death: diabetes, cardiovascular disease, obesity, pulmonary disease, immunocompromised status, rheumatological condition, and cancer. This variable was dichotomized to distinguish the presence or absence of comorbidities.

Participants were asked to select up to three sources of information for COVID-19 vaccines. Possible choices were TV, newspapers, radio, institutional websites, social media, and word of mouth (friends, family, colleagues, etc…). Because the sources were not mutually exclusive, they were treated as dummy variables according to whether they were used or not.

### 2.3. Theoretical Model

In a previous analysis [17] we showed that age, gender, educational level, family income, perceived risk of infection, presence of comorbidities, and previous vaccination refusal were significant determinants of COVID-19 vaccine hesitancy. In this paper we hypothesize that information sources could act as mediators and explain, at least partially, the relationship between individual characteristics and vaccine hesitancy.

### 2.4. Statistical Analysis

Variables were described as absolute and relative frequencies, and the entire set of pairwise correlations was estimated by means of Cramér’s *V*. The association between information source use and hesitancy was assessed by comparing the proportion of hesitant respondents among the users of each source with the proportion of hesitant respondents in the overall sample using the exact binomial test. Sources related to a significant different proportion of hesitant users were then included in the mediation analysis.

A mediation analysis was conducted to test the hypothesized relationships between the variables included in the theoretical model using the structural equation modeling (SEM) statistical technique. In the mediation model, the perceived risk of infection was defined as a latent variable based on the three related questions. Model parameters and standard error estimates were calculated with the diagonal weighted least square (DWLS) estimator for categorical dependent variables [41]. To allow the comparison of effects, results were reported as standardized coefficients (std b). The direct, indirect (mediated), and total effects were calculated.

A model including all plausible relationships between independent and dependent variables was initially estimated. The model was then simplified in subsequent steps, constraining non-significant path coefficients (*p* > 0.05) to 0 and comparing each nested model with the former by means of ANOVA. The significant relationships of the final model were graphically represented in a path diagram where red lines denote positive associations between variables and blue lines denote negative associations.

Goodness of fit of the final parsimonious model to the data was evaluated calculating a series of fit indices: the root mean square error of approximation (RMSEA) is related to the model residuals and is considered good when <0.05; the standardized root mean square residual (SRMR) is an indicator of the residual correlation unexplained by the model, and values < 0.06 are considered acceptable; the Tucker–Lewis index (TLI) and the comparative fit index (CFI) are indicators of the improvement of the estimated model compared to the null model, and values > 0.9 indicate a good fit to the data [42,43].

Finally, modification indices (MIs) were used to explore any relationship between independent variables that was not considered in the theoretical model; relationships and correlations with MI > 10 were evaluated and added to improve model fit when considered appropriate.

Statistical significance was set at alpha < 0.05. Analyses were conducted using R version 4.0.5 (R Core Team (2021). R: A language and environment for statistical computing. R Foundation for Statistical Computing, Vienna, Austria.).

## 3. Results

### 3.1. Sample Characteristics and Association between Information Source Use and Vaccine Hesitancy

The sample included 1011 people (55.2% female). The mean age was 46.9 ± 11.5, ranging from 19 to 70 years old. The characteristics of the respondents are reported in Table 1.

Overall, 68.9% of the sample reported being confident about the vaccine, while 31.1% reported being hesitant. A correlation matrix showing the pairwise correlation coefficients of all of the variables is presented in Supplementary Materials Table S1.

Table 2 shows the number of users of each information source and the proportion of hesitant users. The majority of respondents reported getting COVID-19 vaccine information from TV (71.9%). The other most used sources were newspaper (43.3%) and institutional websites (39.2%). Social media were used by 18.9% of respondents. The proportion of hesitant respondents among traditional media (TV, newspaper, and radio) users and people who reported word of mouth was similar and in line with the proportion in the overall sample (28.1–30.0%). Conversely, among social media users, the proportion of hesitant respondents increased to 40.8% (*p* = 0.005), while it decreased to 24.2% among users of institutional websites (*p* = 0.003).

### 3.2. Mediation Analysis

Based on the significant association found between information sources and vaccine hesitancy, social media use and institutional website use were included as mediators in the mediation analysis.

The direct, indirect, and total effects of determinants and mediators on vaccine hesitancy are reported in Table 3. There was a strong positive relationship between social media use and vaccine hesitancy (std b = 0.179, *p* < 0.001), and a negative relationship between institutional website use and vaccine hesitancy (std b = −0.131, *p* = 0.006). Past vaccination refusal (std b= 0.381, *p* < 0.001), perceived risk of infection (std b = −0.230, *p* < 0.001), and presence of comorbidities (std b = −0.159, *p* < 0.001) were confirmed as significant predictors of vaccine hesitancy. Moreover, the presence of comorbidities was associated with a higher use of both social media and institutional websites resulting in a null indirect effect (std b = 0.005, *p* = 0.694).

An undergraduate educational level and being aged 35–44 years of age (compared to age 55–70 years) have a significant positive and direct effect on vaccine hesitancy (std b = 0.100, *p* = 0.010 and std b = 0.138, *p* = 0.005, respectively). No significant direct effect of being aged 18–34 or 45–54 and family income was found. However, the relationship between these variables and vaccine hesitancy was partially mediated by the use of information sources: in particular, there was a significant, though small, indirect effect of being aged 18–34 and higher social media use (std b = 0.026, *p* = 0.032), an almost significant indirect effect of being aged 45–54 and a lower use of institutional websites (std b = 0.016, *p* = 0.066), and an almost significant and indirect effect of high income and a higher use of institutional websites (std b = −0.012, *p* = 0.077). In the mediation model, female gender was not significantly related, nor directly neither indirectly, to vaccine hesitancy.

**Figure 1:**
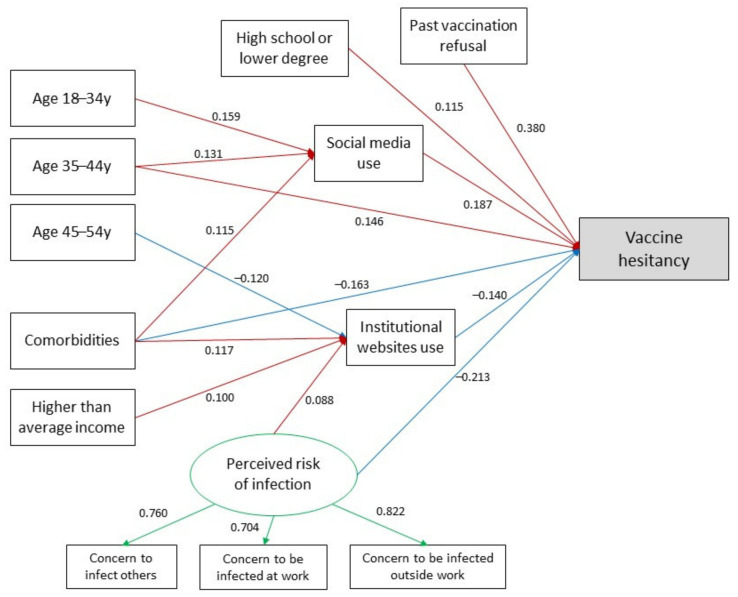
Final mediation model. Red lines denote positive effect and blue lines denote negative effect. Numbers on red and blue arrows represent standardized regression coefficients. Green lines denote the perceived risk of infection latent factor and the relationship with the corresponding observed variables. Numbers on green arrows represent standardized factor loadings.

Figure 1 shows the final parsimonious mediation model with standardized significant regression coefficients. The negative relationships between the age of 45–54 and the use of institutional websites and the positive relationship between higher-than-average income, perceived risk of infection, and the use of institutional websites emerged as significant (*p* < 0.05, see Supplementary Materials Table S2). Similarly, the positive association between the age of 35–44 years and the use of social media was significant (std b = 0.131, *p* = 0.023).

The final mediation model had an acceptable fit to the data, with the exception of CFI, which was below the threshold of 0.9: RMSEA 0.050 (95%CI: 0.041–0.060), SRMR 0.044, TLI 0.913, CFI 0.798. By means of the MI indices, the SEM model was amended with the addition of perceived risk of infection as a mediator and the addition of the correlation between social media and institutional websites. The effect of age and the presence of comorbidities were also mediated by the perceived risk of infection. These additional pathways led to an improvement in the model’s fit indices, resulting in a CFI close to the threshold (CFI 0.906) (see Supplementary Materials Table S3).

## 4. Discussion

In this paper, we found that the use of social media directly increased or mediated the risk of vaccine hesitancy towards COVID-19 vaccination, while the opposite was found for institutional websites. Social media are now an integral part of our daily lives, and it is not possible to ignore their huge role in communication and in interactions among people. In the last decade, there has been a tremendous rise in the use of social media, which allow people to create, share, or exchange information in virtual communities and networks [30]. Debate on social media has been shown to be dominantly anti-vax, with the majority of vaccine-related content expressing negative views and relaying false information [34,35]. Our findings could be related to the previously mentioned apparent advantage of content expressing hesitant views to the intentional and eased dissemination of false information with the aim of fuelling vaccine hesitancy and to the amplification of discussions that can create the misleading perception of a debate that is not actually taking place in the scientific community.

Social media use can mediate the risk of vaccine hesitancy as observed for the age classes of 18–34 and 35–44 years of age in comparison to the age class of 55–70 years of age. Moreover, belonging to age class of 35–44 years of age can directly increase the risk of vaccine hesitancy; participants in the 45–54 age class were found to use institutional websites less that the oldest age class. People aged 55 years of age or more could use institutional website more than the younger age classes because of the awareness of potentially being at higher risk of symptomatic disease, thus searching for verified information.

People with comorbidities had a direct decreased risk of vaccine hesitancy, and this could be related to a higher risk perception and awareness of potential severe COVID-19 and poor outcomes among patients affected by chronic disease [44,45,46,47]. Interestingly, they relied both on social media and institutional websites as a source of information on vaccination towards COVID-19, and this could be probably due to a profound need of information in relation to their condition or to a possible higher confidence in official scientific sources because of their frequent contact with healthcare personnel and a possible greater sensitivity to vaccination to protect themselves from the burden of SARS-CoV-2 infection.

Participants that declared an income higher than the average were more likely to use institutional websites, thus also had a reduced risk of vaccine hesitancy. In the literature, a lower income has been associated with a higher risk of hesitancy [48].

Lower educational level and past vaccination refusal were not mediated by either the use of social media or by the use of institutional websites, and for both, an increased risk of vaccine hesitancy has been shown, as previously pointed out in the literature [17,49,50,51,52].

Finally, a higher perceived risk of infection determined a reduced risk of vaccine hesitancy both directly and mediated by the use of institutional websites. This could be explained considering that vaccination could be seen as a valuable tool against the disease and that people with higher risk perception could refer to official guidelines to seek measures to avoid contagion.

Compared to previous analysis [17], we found that the significant effect of age (45–54 years) and income was mediated by the use of institutional websites. However, for the other variables, the indirect effect was relatively small compared to the direct effect; in other words, the use of information sources can only partially explain the relationship between individual characteristics and vaccine hesitancy. This does not mean that information sources are irrelevant in determining vaccine acceptance or hesitancy, rather, their effect adds up to the direct effect of other variables.

Our study has some limitations. The survey was conducted at the beginning of the vaccination campaign; therefore, in the meantime, attitudes toward COVID-19 vaccinations may have varied. As this is a cross-sectional study, causal relationships could not be inferred. Nevertheless, associations between variables should be considered as suggestive of possible cause–effect relationships. Data were collected in a specific geographical area corresponding to the Emilia-Romagna region; thus, results cannot be generalized to the entire Italian population. However, as the individual regions are responsible for healthcare provision in Italy, the results of this study provide evidence to local stakeholders and institutions that could be useful for promoting concrete actions. The age of the respondents was limited to 18–70 years old due to the nature of data collection through an online platform; thus, the results cannot be generalized to the overall population. This paper did not evaluate the frequency of use of each information source, which perhaps could determine its effect on individual attitudes. Moreover, using and dichotomizing the Likert scale has limitations in capturing in depth reasons for vaccine hesitancy. Finally, it was not possible to conduct sensitivity analyses by altering the number of measurement units of categorical data to evaluate the robustness of our mediation model, and no formal preliminary reliability assessment of our instrument was performed [53].

Our findings highlight the role of information sources in relation to COVID-19 vaccination and some considerations might emerge.

Multiple sources and the ease and speed with which information is constantly updated and corrected could create disorientation and distrust in people who are not against vaccination but who simply seek for further information and answers on social media. Therefore, it is important to take a great value of social media into account, which is the possibility of creating networks: this property could be exploited to create networks of professionals that share common verified content and that collectively represent a unique and compact voice to increase trust on which people can rely. Social media holds considerable unexpressed potential in helping contain vaccine hesitancy: invaluable instruments such as real-time fact-checking, the direct interaction with experts in the field and policymakers, and the possibility of easily redirection towards official and reliable information sources could help tackle health illiteracy on the matter. Therefore, their use should be enhanced to expand the audience and to maximize their potentially positive impact. However, enhancing their use with the purpose of disseminating scientifically sound and verified information is not enough. Measuring the outcomes of strategies based on these tools is essential to make adjustments and to increase the effectiveness of the efforts.

Moreover, transparency and widespread diffusion of information on the processes behind vaccine approval and distribution could be useful in tackling mistrust in institutions. It is also essential to investigate the characteristics of social media users and the most currently used social media in order to produce targeted content, considering that the way in which people connect to each other varies hugely across different social media and that the social media landscape itself could rapidly change [54].

However, social media should not be intended as a substitute for broad-based communication strategies, and their adoption should not take energy and resources away from more traditional health promotion activities. Our results showed that people rely on institutional websites and specifically that their use can reduce vaccine hesitancy towards COVID-19 vaccination. Considering their positive impact, it could be useful to strengthen their connection with social media by inserting labels that refer users to official sources matching social media content related to vaccines, a strategy already adopted by Instagram and Twitter [55,56]. Institutions and public health organizations could land on even more social media, with official profiles embracing a style of communication in line to the different social media and through advertisement.

Both traditional channels and social media could be helpful in engaging different groups regarding public health policies and counter misinformation [19], thus contributing to counteract vaccine hesitancy.

## Figures and Tables

**Table 1:** Characteristics of the sample (N = 1011).

Characteristics	N (%)
**Gender**	
Male	453(44.8)
Female	558 (55.2)
**Age (years)**	
18–34	154 (15.2)
35–44	271 (26.8)
45–54	314 (31.1)
≥55	272 (26.9)
**Education**	
High school or lower degree	642 (63.5)
Bachelor’s or higher degree	369 (36.5)
**Family income**	
Higher than average	104 (10.3)
On average	591 (58.5)
Lower than average	316 (31.3)
**Comorbidities**	
No	724 (71.6)
One or more	287 (28.4)
**Past vaccination refusal**	
No	853 (84.4)
Yes	158 (15.6)
**Concern about contracting COVID-19 at work**	
Not at all	182 (18.0)
A little	308 (30.5)
Somewhat	346 (34.2)
Very concerned	175 (17.3)
**Concern about contracting COVID-19 outside of work**	
Not at all	88 (8.7)
A little	298 (29.5)
Somewhat	434 (42.9)
Very concerned	191 (18.9)
**Concern about infecting family or friends with COVID-19**	
Not at all	56 (5.5)
A little	151 (14.9)
Somewhat	376 (37.2)
Very concerned	428 (42.3)
COVID-19 vaccine hesitancy	
No	697 (68.9)
Yes	314 (31.1)

**Table 2:** Frequency of use of each information source and proportion of hesitant users.

Source	Users N (%)	Hesitant Users N (%)	*p*-Value
TV	727 (71.9%)	211 (29.0%)	0.245
Newspapers	438 (43.3%)	123 (28.1%)	0.197
Radio	90 (8.9%)	27 (30.0%)	0.910
Institutional websites	396 (39.2%)	96 (24.2%)	0.003
Social media	191 (18.9%)	78 (40.8%)	0.005
Word of mouth	182 (18.0%)	53 (29.1%)	0.631

Note: percentage of users is computed overall (N = 1011); percentage of hesitancy is computed among users of each source.

**Table 3:** Standardized direct, indirect, and total effects on vaccine hesitancy.

	Direct Effect		Indirect Effect via Institutional Websites		Indirect Effect via Social Media		Total Effect	
	Estimate	*p*-Value	Estimate	*p*-Value	Estimate	*p*-Value	Estimate	*p*-Value
**Individual determinants**								
Female gender	0.047	0.230	−0.003	0.611	0.010	0.243	0.054	0.161
Age (reference category ≥55 y)								
18–34 y	−0.025	0.607	0.001	0.881	**0.026**	**0.032**	0.003	0.954
35–44 y	**0.138**	**0.005**	0.007	0.332	0.021	0.080	**0.166**	**0.001**
45–54 y	0.062	0.195	0.016	0.066	0.012	0.285	0.090	0.060
High school or lower degree	**0.100**	**0.010**	0.002	0.730	0.013	0.161	**0.115**	**0.003**
Higher than average family income	−0.054	0.207	−0.012	0.077	−0.006	0.522	−0.072	0.088
Presence of comorbidities	**−0.164**	**<0.001**	**−0.015**	**0.047**	**0.020**	**0.042**	**−0.159**	**<0.001**
Past vaccination refusal	**0.373**	**<0.001**	0.005	0.350	0.003	0.716	**0.381**	**<0.001**
Perceived risk of infection	**−0.214**	**<0.001**	−0.012	0.088	−0.005	0.595	**−0.230**	**<0.001**
**Mediators**								
Social media use	**0.179**	**<0.001**					**0.179**	**<0.001**
Institutional websites use	**−0.131**	**0.006**					**−0.131**	**0.006**

Significant estimates and corresponding *p*-values in bold.

## Data Availability

All data have been stored and kept by E.M. and M.P.F., who were responsible for data analysis and the appropriate keeping of the data. Original data are available upon request to the corresponding author.

## Supplementary Materials

The following are available online at https://www.mdpi.com/article/10.3390/idr13030066/s1, File S1: Questionnaire instrument, Table S1: Correlation matrix of all variables considered for inclusion in the mediation model. Pairwise correlations are expressed by means of Cramer’s V. Table S2: Standardized regression coefficients from final mediation model, Table S3: Standardized regression coefficients from final mediation model with the addition of perceived risk of infection as a mediator.
