# Supplementary material for: Vaccine Hesitancy towards COVID-19 Vaccination: Investigating the Role of Information Sources through a Mediation Analysis
Source: Infect Dis Rep. 2021 Aug 12;13(3):712–23. doi: 10.3390/idr13030066 (PMC8395997; doi:10.3390/idr13030066)
Supplement: Supplementary file 1 [file idr-13-00066-s001.zip › idr-1300922-supplementary.pdf]

Questionnaire instrument

Panel information already available:

- Age (years)
- Gender
- Educational level
- Employment status
- Number of family members

1. You would define your family income as…

- o Lower than average
- o On average
- o Higher than average

2. Do you have any of the following conditions? [select all that apply]

- o Cancer
- o Immunocompromised state due to therapy or disease
- o Obesity
- o Diabetes (type 1 or 2)
- o Cardiovascular disease
- o Pulmonary disease
- o Rheumatological condition

3. If you have refused a vaccine in the past that was recommended to you by a healthcare worker - what was/were the reason(s)? [check all reasons that applied to that situation]

- o I never refused a vaccine recommended by a healthcare worker
- o Did not think it was needed
- o Did not have enough information on the vaccine
- o Did not think the vaccine was effective
- o Did not think the vaccine was safe
- o I was concerned about side effects
- o I had a bad experience with a previous vaccination
- o Did not know where to get vaccination
- o Other logistic problems

4. How concerned are you of ...

	Not at all concerned	A little concerned	Somewhat concerned	Very concerned
Contracting COVID-19 at work?<br>(For example: office and other work settings that are not your home)				
Contracting COVID-19 outside of work?<br>(For example: at the grocery store, when you are using transportation, or in other aspects of your daily life)				
Infecting your family or friends with COVID-19?				

5. A COVID-19 vaccine has already been approved. If you were offered to get the vaccine in the next months - at no cost for you- how likely are you to take it?

- Very likely
- Somewhat likely
- I am not sure
- Somewhat unlikely
- Very unlikely
- I would not take it within the next two months but I might reconsider it in the future

6. Which information sources have you mostly used to get information about the COVID-19 vaccine? Select up to 3 sources.

- TV
- Newspaper
- Radio
- Institutional websites
- Social Media
- Word of mouth (familiars, friends, colleagues…)

Supplementary Results

Table S1. Correlation matrix of all variables considered for inclusion in the mediation model. Pairwise correlations are expressed by means of Cramér's *V*.

	Gender	Age group	High school or lower degree	Higher than average family income	Presence of comorb.	Past vaccination refusal	Concerns about getting infected at work	Concerns about getting infected outside of work	Concerns about infecting dear ones	TV as inform. source	Newspapers as inform. source	Radio as inform. source	Social media as inform. source	Institutional websites as inform. source	Word of mouth as inform. source	Vaccine hesitancy
Gender	1.000															
Age group	0.202***	1.000														
Education	0.014	0.151***	1.000													
Income	0.112**	0.068	0.214***	1.000												
Comorbidities	0.006	0.226***	0.058	0.090*	1.000											
Past refusal	0.061	0.113**	0.004	0.139***	0.043	1.000										
Infected at work	0.088*	0.086**	0.072	0.077	0.069	0.075	1.000									
Infected elsewhere	0.127***	0.034	0.017	0.115***	0.093*	0.061	0.514***	1.000								
Infecting dear ones	0.147***	0.116***	0.047	0.066	0.060	0.095*	0.386***	0.460***	1.000							
TV	0.050	0.065	0.066*	0.078*	0.036	0.107**	0.048	0.107**	0.073	1.000						
Newspapers	0.067*	0.068	0.088**	0.160***	0.007	0.008	0.026	0.068	0.060	0.049	1.000					
Radio	0.061	0.069	0.008	0.059	0.027	0.001	0.072	0.029	0.033	0.037	0.063*	1.000				
Social media	0.059	0.082	0.046	0.039	0.060	0.001	0.042	0.022	0.056	0.103**	0.131***	0.036	1.000			
Institutional sites	0.010	0.096*	0.019	0.089*	0.097**	0.027	0.068	0.077	0.076	0.175***	0.023	0.101**	0.087**	1.000		
Word of mouth	0.106**	0.068	0.013	0.021	0.010	0.032	0.045	0.052	0.104	0.074*	0.145***	0.047	0.042	0.118***	1.000	
Hesitancy	0.037	0.119**	0.078*	0.114**	0.110***	0.329***	0.114***	0.188***	0.191***	0.070*	0.056	0.007	0.102**	0.118***	0.020	1.000

$^{***}p$-value ≤0.001; $^{**}p$-value ≤0.01; $^{*}p$-value ≤0.05.

Table S2. Standardized regression coefficients from final mediation model.

	Vaccine hesitancy		Institutional websites use		Social media use	
	estimate	p-value	estimate	p-value	estimate	p-value
Age (reference category ≥55y)						
18-34y	-0.018	0.707	-0.003	0.944	**0.159**	0.003
35-44y	**0.146**	0.003	-0.047	0.333	**0.131**	0.023
45-54y	0.064	0.186	**-0.120**	0.013	0.071	0.227
High school or lower degree	**0.115**	0.003	-	-	-	-
Higher than average family income	-	-	**0.100**	0.013	-	-
Presence of comorbidities	**-0.163**	<0.001	**0.117**	0.003	**0.115**	0.013
Past vaccination refusal	**0.380**	<0.001	-	-	-	-
Perceived risk of infection	**-0.213**	<0.001	**0.088**	0.040	-	-
Social media use	**0.187**	<0.001				
Institutional websites use	**-0.140**	0.004				

In bold significant coefficients. Fit indices: robust CFI=0.798, robust TLI=0.913, robust RMSEA=0.050 (95%CI: 0.041 – 0.060), SRMR=0.044.

Table S3. Standardized regression coefficients from final mediation model with the addition of perceived risk of infection as a mediator.

	Vaccine hesitancy		Institutional websites use		Social media use		Perceived risk of infection	
	estimate	p-value	estimate	p-value	estimate	p-value	estimate	p-value
Age (reference category ≥55y)								
18-34y	0.021	0.666	-0.018	0.703	**0.159**	0.003	**0.161**	<0.001
35-44y	**0.173**	<0.001	-0.056	0.250	**0.131**	0.023	**0.097**	0.022
45-54y	**0.093**	0.049	**-0.130**	0.007	0.071	0.227	**0.110**	0.009
High school or lower degree	**0.116**	0.003	-	-	-	-	-	-
Higher than average family income	-	-	**0.099**	0.014	-	-	-	-
Presence of comorbidities	**-0.141**	0.001	**0.107**	0.007	**0.115**	0.013	**0.108**	0.003
Past vaccination refusal	**0.382**	<0.001	-	-	-	-	-	-
Perceived risk of infection	**-0.220**	<0.001	**0.091**	0.036	-	-		
Social media use	**0.168**	0.002						
Institutional websites use	**-0.112**	0.026						

In bold significant coefficients. Correlation between social media use and institutional websites use: r= -0.173, p=0.002. Fit indices: robust CFI=0.906, robust TLI=0.953, robust RMSEA=0.037 (95%CI: 0.026 – 0.048), SRMR=0.028.
